# Training-induced changes in population receptive field properties in visual cortex: Impact of eccentric vision training on population receptive field properties and the crowding effect

**DOI:** 10.1167/jov.24.5.7

**Published:** 2024-05-21

**Authors:** Maka Malania, Yih-Shiuan Lin, Charlotte Hörmandinger, John S. Werner, Mark W. Greenlee, Tina Plank

**Affiliations:** 1 Institute of Psychology, University of Regensburg, Regensburg, Germany; 2 Department of Ophthalmology and Vision Science, University of California, Davis, Sacramento, CA, USA

**Keywords:** pRF, brain plasticity, crowding, perceptual learning, retinotopic mapping, fMRI

## Abstract

This study aimed to investigate the impact of eccentric-vision training on population receptive field (pRF) estimates to provide insights into brain plasticity processes driven by practice. Fifteen participants underwent functional magnetic resonance imaging (fMRI) measurements before and after behavioral training on a visual crowding task, where the relative orientation of the opening (gap position: up/down, left/right) in a Landolt C optotype had to be discriminated in the presence of flanking ring stimuli. Drifting checkerboard bar stimuli were used for pRF size estimation in multiple regions of interest (ROIs): dorsal-V1 (dV1), dorsal-V2 (dV2), ventral-V1 (vV1), and ventral-V2 (vV2), including the visual cortex region corresponding to the trained retinal location. pRF estimates in V1 and V2 were obtained along eccentricities from 0.5° to 9°. Statistical analyses revealed a significant decrease of the crowding anisotropy index (*p* = 0.009) after training, indicating improvement on crowding task performance following training. Notably, pRF sizes at and near the trained location decreased significantly (*p* = 0.005). Dorsal and ventral V2 exhibited significant pRF size reductions, especially at eccentricities where the training stimuli were presented (*p* < 0.001). In contrast, no significant changes in pRF estimates were found in either vV1 (*p* = 0.181) or dV1 (*p* = 0.055) voxels. These findings suggest that practice on a crowding task can lead to a reduction of pRF sizes in trained visual cortex, particularly in V2, highlighting the plasticity and adaptability of the adult visual system induced by prolonged training.

## Introduction

Although changes in the brain reflecting neuroplasticity are most evident during early development (Berardi, Pizzorusso, & Maffei, 2000; Espinosa & Stryker, 2012; Hubel & Wiesel, 1965; Hubel, Wiesel, & LeVay, 1977; Mioche & Singer, 1989), human brains continue to be shaped by experience to adjust to environmental changes. Separate neural systems have different demands for plasticity. Sensory systems that are responsible for the continuous adjustment to the changing of input statistics maintain a high degree of plasticity throughout the lifespan (Baylor, 1987; Dunn, Lankheet, & Rieke, 2007; Stiles, 2000; Wade & Wandell, 2002; Wandell & Smirnakis, 2009). Visual perceptual learning refers to repeated practice on the visual task that induces long-term improvement in visual performance (Gilbert, Sigman, & Crist, 2001; Sasaki, Nanez, & Watanabe, 2010). Evidence for adult brain plasticity has been demonstrated by behavioral and neural changes associated with visual perceptual learning (Beyeler, Rokem, Boynton, & Fine, 2017; Dosher & Lu, 2017; Fahle & Poggio, 2002; Fiorentini & Berardi, 1980; Watanabe & Sasaki, 2015). Moreover, these changes can be long-lasting, indicating the persistence of plasticity effects (Frank, Greenlee, & Tse, 2018).

In this study, we addressed the issue of adult brain plasticity in early visual cortex resulting from training on a visual crowding task. Visual crowding refers to the impaired recognition of eccentrically presented objects when they are surrounded by clutter (Bouma, 1973; Flom, Weymouth, & Kahneman, 1963; Levi, 2008). The strength of visual crowding is influenced by various factors, including the spatial arrangement of the flanking stimuli. Specifically, radial flankers cause a more pronounced crowding effect than tangentially aligned flankers. This phenomenon is known as the anisotropy of visual crowding (Kwon, Bao, Millin, & Tjan, 2014; Toet & Levi, 1992; Whitney & Levi, 2011).

There is evidence that the strength and extent of visual crowding can be reduced by training (Chung, 2007; Hussain, Webb, Astle, & McGraw, 2012; Plank et al., 2021; Sun, Chung, & Tjan, 2010; Wolford, Marchak, & Hughes, 1988). In our earlier study, we demonstrated a significant positive correlation between changes in the crowding anisotropy index and changes in the blood oxygen level–dependent (BOLD) response within the brain region corresponding to the trained visual field (Malania, Pawellek, Plank, & Greenlee, 2020). However, the specific neural modifications associated with the performance improvement in the crowding task are yet to be determined.

Previous research has indicated the potential involvement of receptive field properties in the emergence and dynamics of visual crowding (Altman & Das, 1965; Flom et al., 1963; He, Cavanagh, & Intriligator, 1996; Parkes, Lund, Angelucci, Solomon, & Morgan, 2001; Pelli, Palomares, & Majaj, 2004; Polat & Sagi, 1993; Strasburger & Malania, 2013). According to the receptive field theory, when stimulus information about both target and flankers falls within a single population receptive field (pRF), that information becomes integrated or pooled together. Consequently, smaller pRFs would decrease the likelihood of target and flankers falling within the same pRF, resulting in weaker crowding effects. He, Wang, and Fang (2019) provided preliminary evidence for this notion. While measuring pRF sizes during the crowding task, they found a correlation between receptive field size and the magnitude of crowding.

To explore the neuronal basis of improvement in performance on a crowding task following perceptual learning, we used a combination of psychophysical techniques and functional magnetic resonance imaging (fMRI). Specifically, this study aimed to estimate pRF properties in early visual cortex to identify potential neural changes associated with training on a crowding task. We implemented a pRF mapping technique (Dumoulin & Wandell, 2008; Smith, Singh, Williams, & Greenlee, 2001) that has recently emerged as a popular tool in human neuroimaging. It provides valuable insights into visual and cognitive processes (Harvey, Dumoulin, Fracasso, & Paul, 2020; He et al., 2019; Klink, Chen, Vanduffel, & Roelfsema, 2021; Poltoratski, Maier, Newton, & Tong, 2019; Shen, Han, & de Lange, 2020; Silson, Reynolds, Kravitz, & Baker, 2018; Stoll, Finlayson, & Schwarzkopf, 2020; Welbourne, Morland, & Wade, 2018), brain dysfunctions (Ahmadi et al., 2020; Alvarez et al., 2020; De Best et al., 2019; Dumoulin & Knapen, 2018; Schwarzkopf, Anderson, de Haas, White, & Rees, 2014), and brain development (Dekker, Schwarzkopf, de Haas, Nardini, & Sereno, 2019).

Building upon our earlier findings and the existing literature on practice-induced brain plasticity, we predict that practice on the visual crowding task will modulate pRF properties in early visual cortex. We hypothesized that the training-related improvement in crowding task performance and the subsequent reduction of the critical spacing can be attributed to a decrease in pRF sizes, with the most pronounced effects observed in the projection zone of the trained visual area.

Overall, these results contribute to our understanding of adult brain plasticity in response to visual perceptual learning, specifically focusing on changes in pRFs in the early visual cortex. By understanding the underlying neural mechanisms associated with these changes, we can gain insights into the plasticity of the visual system and its implications for visual perception.

## Methods

### Participants

We recruited 17 participants (11 females and six males) with normal or corrected-to-normal visual acuity who reported no history of psychiatric or neurological disorders. All participants were right-handed. The age of the participants ranged from 21 to 40 years, with a mean age of 25.3 years and *SD* of 5.05. Two subjects were excluded from the final statistical analysis due to difficulties in maintaining central fixation and consequently their failure to perform the central fixation task during fMRI scans. Thus, the data analysis is based on results from 15 participants (10 females and five males). All participants provided written informed consent and received course credits for their participation but otherwise no financial compensation. The study was approved by the University of Regensburg research ethics committee and conducted in accordance with the tenets of the Declaration of Helsinki.

### General procedure

The study involved two MRI measurements conducted before and after psychophysical training to assess the average size of the pRFs in early visual cortex. Additionally, separate runs were carried out to determine the retinotopic maps of V1, dV2, and vV2 using established wedge and ring stimuli (DeYoe et al., 1996; Dumoulin & Wandell, 2008; Engel et al., 1994; Sereno et al., 1995). Moreover, T1-weighted images of the gray and white matter of the entire brain were acquired. Finally, an additional fMRI run was performed to identify the specific region of the visual cortex that experienced stimulation during the training on the visual crowding task. This ROI will be referred to as the trained ROI (tROI).

### Psychophysical experiments

#### Stimuli and experimental procedure

**Figure 1.**
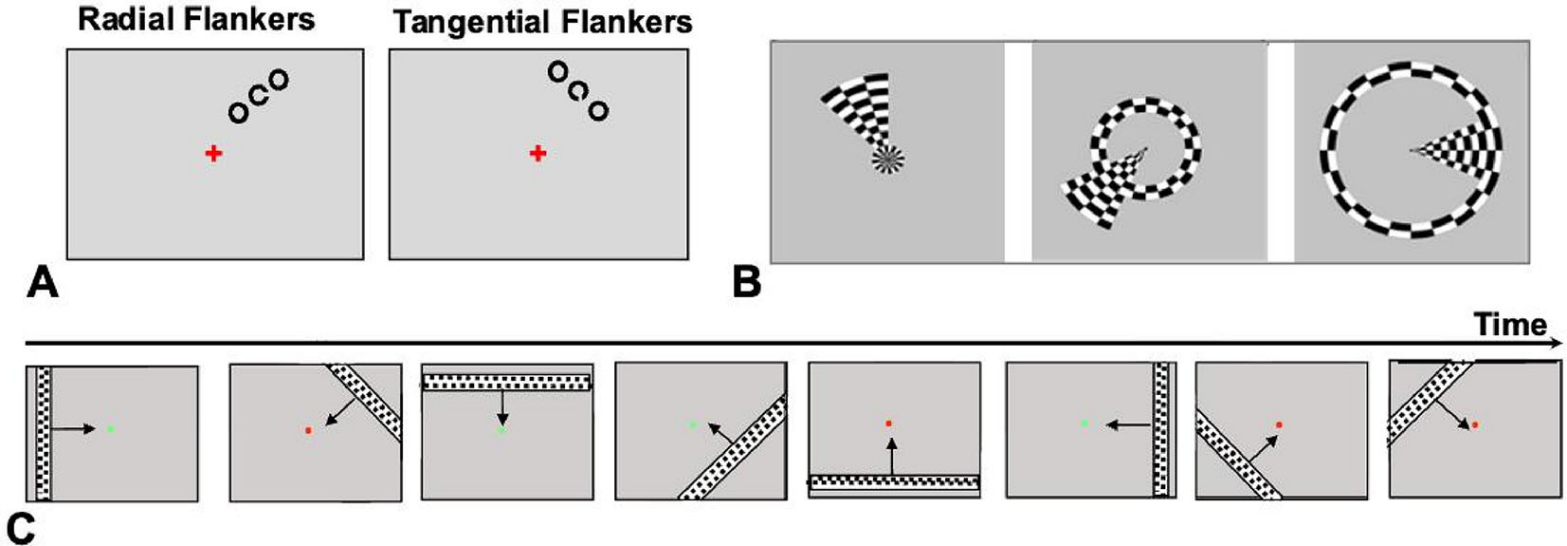
Illustration of experimental stimuli. (**A**) Examples of stimuli used in psychophysical experiments. The stimuli consisted of a Landolt C surrounded by rings of equal size. Each training block presented a single flanker configuration, either radial or tangential. (**B**) Rotating wedges and ring stimuli were used in the retinotopic mapping experiments at three exemplary time points. These stimuli were used to map the visual field in V1, dV2, and vV2 regions. (**C**) Examples of moving-bar stimuli used to estimate the pRF properties. The bars moved in one of eight different directions across the entire screen during the stimulation blocks, which were interchanged with fixation blocks consisting of a blank screen with only a fixation point. The arrows merly depict the motion of the bars and were not presented during the experiment.

In the psychophysical experiments, stimuli were presented on a liquid-crystal display monitor with a resolution of 1024 × 768 pixels and refresh rate of 75 Hz. The monitor had a screen size of 37.5 × 30 cm. The head of each participant was supported by a chin rest to maintain a constant viewing distance of 54 cm. The stimuli were presented and generated by using Presentation 17.0 software (Neurobehavioral Systems, Berkeley, CA). The target stimulus consisted of a high-contrast Landolt C, flanked by two same-sized rings positioned either radially or tangentially with respect to central fixation. Both the target and flankers had a size of 0.75 degree visual angle. The stimuli were presented in the right upper visual quadrant, specifically 25° clockwise from the vertical meridian at an eccentricity of 6.5°. Stimuli stayed on the screen for 67 ms, followed by a gray screen presented for 200 ms. A small fixation cross was presented in the center of the screen. Example stimuli used in psychophysical experiments are illustrated in Figure 1A. The luminance of the monitor was gamma corrected using a Minolta spot photometer (Konica Minolta, Tokyo, Japan). The background luminance was ∼151.6 cd/m^2^, and the stimulus luminance was ∼0.2 cd/m^2^ (Weber contrast, ∼0.99).

The behavioral training took place on four consecutive days, with two training blocks per day, separately for tangential and radial flanker configurations. Prior to training on the crowding task, participants underwent a short practice session to familiarize themselves with the task. Each training block was divided into two sub-blocks, each lasting approximately 25 minutes, during which participants completed 432 trials. Consequently, subjects were presented with each flanker condition a total of 864 times. On each trial, subjects were presented with a Landolt C surrounded by two same-sized rings, arranged either radially or tangentially relative to the central fixation. The orientation of the gap in the Landolt C varied randomly among up, down, right, or left on each trial. Furthermore, the target-to-flanker spacing was varied randomly between 0.75° and 3° with a step size of 0.5° using the method of constant stimuli. Subjects were instructed to maintain central fixation and to indicate the gap direction in the Landolt C using a four-alternative, forced-choice task. They provided their responses by pressing the corresponding arrow keys on a computer keyboard. The magnitude of the crowding effect was quantified as the critical spacing, defined as the minimum distance required between a target and flankers to allow for target recognition.

### MRI experiments

#### MRI settings

The MRI images were collected with a 3T Prisma scanner (Siemens, Erlangen, Germany) with a 64-channel head coil. High-resolution T1-weighted structural images were obtained with a gradient-echo sequence, acquiring 176 slices with 1 × 1 × 1-mm isotropic voxels. The acquisition parameters included a flip angle of 9°, an echo time of 2.6 ms, and a repetition time of 2.3 seconds. The functional T2*-weighted images were acquired with an echo planar imaging sequence at a flip angle of 90° and at an echo time of 30 ms. The repetition time was set to 2 seconds for the tROI determination and the pRF experiments and 1.5 seconds for the retinotopic mapping experiments. The voxel size was 2 × 2 × 2 mm.

All visual stimuli were backprojected onto a translucent screen (40 × 30 cm; resolution, 1024 × 768 pixels; refresh rate, 60 Hz) using a calibrated PROPixx projector (VPixx Technologies, Saint-Bruno-de-Montarville, QC, Canada). The luminance value for the background was calibrated to ∼171.5 cd/m^2^, and the stimulus luminance for the Landolt C localizer stimuli was ∼0.19 cd/m^2^. The subjects viewed the stimuli via a mirror that was mounted to the head coil, and the viewing distance was 95 cm.

#### Retinotopic mapping

For the retinotopic mapping, stimuli were generated by adapting a MATLAB script (MathWorks, Natick MA) based on Psychtoolbox. The codes are available in the VISTASOFT software repository (https://vistalab.stanford.edu/software). We combined polar angle and eccentricity mapping procedures into a single session. For the polar angle mapping, a high contrast (∼99%) checkerboard pattern moving in the shape of rotating wedges stepping through a clockwise direction was used. The wedge subtended an area of 45° polar angle. Simultaneously, a checkerboard pattern moved in the shape of an expanding or contracting ring to map eccentricity. The ring had a width of 1.5° and covered the visual field from central (0.2°) to eccentric (9°) locations. The pattern was flickering in counterphase at 4 Hz. A small black fixation dot was presented at the center of the screen, and participants were instructed to maintain central fixation throughout the measurement. Examples of the combined retinotopic stimuli are shown in Figure 1B.

One full cycle of the ring stimuli was comprised of 12 steps while the wedges were sequentially shifted by one of 16 steps with each step corresponding to a 22.5° polar angle. (stimulus duration was 3 seconds). The wedges completed one full cycle within 48 seconds, whereas a full cycle (expansion and contraction) of the rings took place within 36 seconds. A run consisted of six cycles for the wedges and eight cycles for the rings. The asynchrony of the stimulus cycles allowed the effects of polar and eccentricity mapping stimuli to be separated.

#### Population receptive field mapping

For the pRF mapping (Dumoulin & Wandell, 2008), a high-contrast (99%) checkerboard pattern in the form of moving bars was used (see Figure 1C for the example stimuli). The visual stimuli were generated by the Psychtoolbox. The bar width was equal to 1/4 of the stimulus radius (9°). The drifting bar moved along eight different directions: horizontal left to right, horizontal right to left, vertical bottom to top, and vertical top to bottom. When the bar was in a vertical or horizontal orientation, it covered the entire screen, whereas in the other orientations it covered only half of the screen. The direction of moving bars was reversed halfway through the presentation of each bar. A small dot was simultaneously presented at the center of the screen. Subjects performed the central fixation task by pressing the button in response to the change of fixation dot color from red to green. The measurements were repeated four times in pre-training and four times in post-training MRI sessions to ensure reliable data.

#### ROI localizer for the crowding task

During the pre-training MRI session, we conducted separate fMRI scans to identify the functional ROI in the visual cortex that corresponded to the stimulated visual field while subjects were trained on the crowding task. As mentioned above, this area is referred to as the tROI.

**Figure 2.**
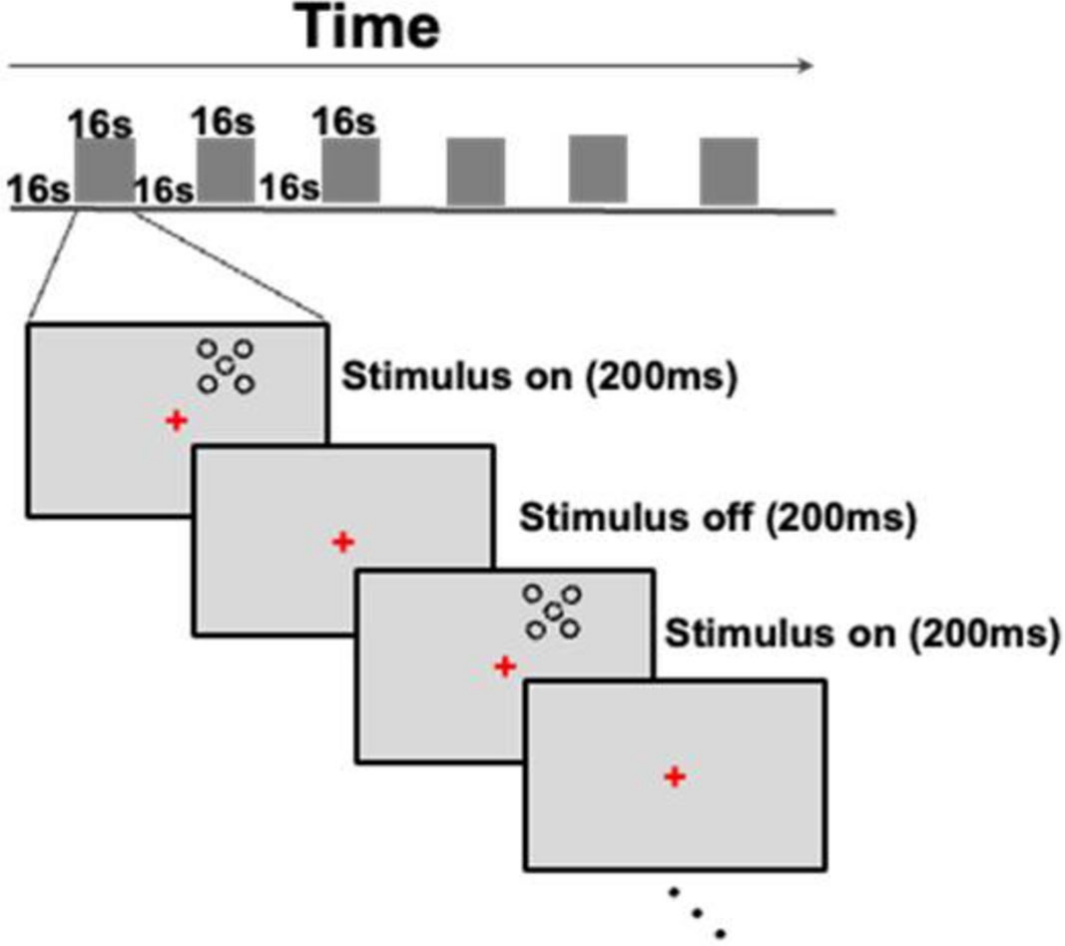
Experimental design and procedure for localizing tROI showing the time course of a typical fMRI run with a central fixation task. We used a block design paradigm in which each fixation block (16 seconds) was followed by a stimulation block (16 seconds). The stimulus displays are not drawn to scale.

Similar to the psychophysical experiment, we used Landolt C target stimuli and the same-sized rings as flankers. The size and position of stimuli were identical to those used during training on the crowding task; that is, stimuli were presented at 6.5° eccentricity in the upper right visual field with a size of 0.75°. However, in this fMRI experiment, we kept the flanker-to-target distance fixed at 0.95° instead of varying it. The stimuli and experimental paradigm are shown in Figure 2.

During the fMRI scans, subjects were asked to maintain central fixation and perform a central fixation task while the crowding stimuli were presented in the periphery. The subjects’ task was to detect a brief and random color change of the fixation cross. They reported this change by pressing one of two buttons. This paradigm allowed us to identify the specific region in the visual cortex that was functionally responding to the crowding stimuli.

### MRI data preprocessing

#### Structural images

The T1-weighted structural images were processed for cortical reconstruction and segmentation using FreeSurfer 6.0 (Athinoula A. Martinos Center for Biomedical Imaging, Boston, MA). The anatomical data processing pipeline involved several steps, including motion correction, removal of non–brain tissue (Ségonne et al., 2004), transformation to Talairach space (Talairach & Tournoux, 1988), intensity normalization, segmentation of the subcortical white matter and deep gray matter (Desikan et al., 2006), and finally volumetric reconstruction and parcellation (Fischl et al., 2004; Fischl, 2012). At each stage of the pipeline, the reconstructed datasets were visually inspected to identify and correct any segmentation errors.

#### Retinotopic mapping

**Figure 3.**
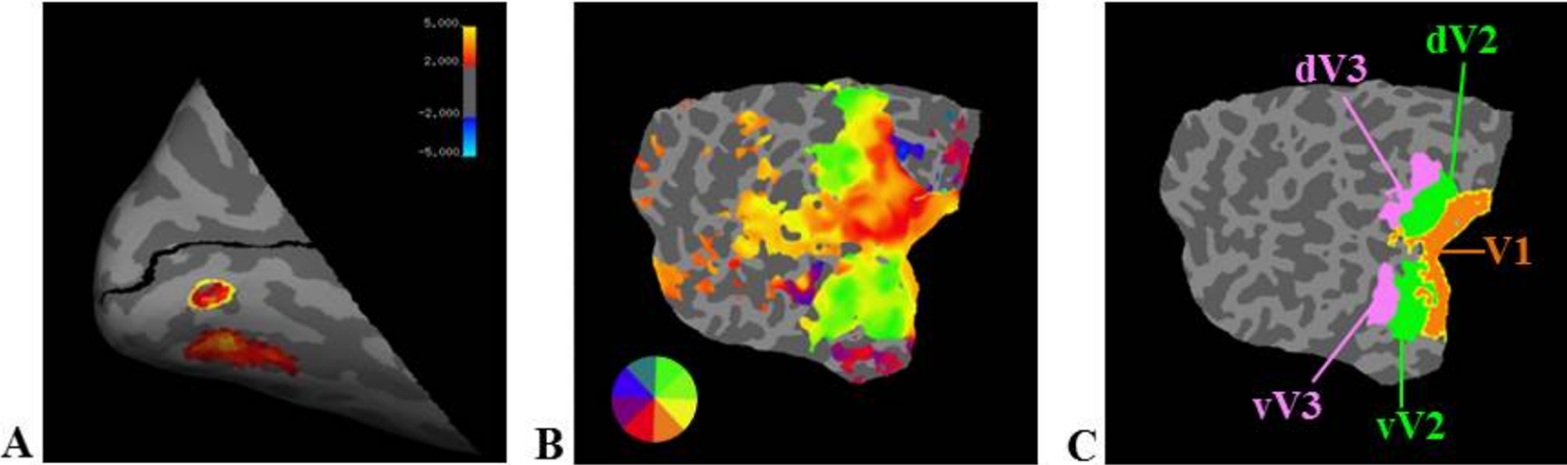
Labeling the ROIs. (**A**) The left inflated occipital cortex displays tROI label for one representative subject. The tROI represents the brain area stimulated during training on the crowding task, and it contained approximately 373 voxels on average. (**B**, **C**) Phase encoding maps (**B**) and delineated ROIs (**C**) for one representative subject.

The visual areas dorsal-V1 (dV1), ventral-V1 (vV1), dorsal-V2 (dV2), and ventral-V2 (vV2) were delineated based on the data from the retinotopic mapping experiments using FreeSurfer 6.0 and custom MATLAB scripts. Initially, the occipital pole was cut along the calcarine fissure and flattened. The acquired retinotopic mapping data underwent preprocessing, including motion correction, brain-mask creation, spatial smoothing, and intensity normalization. Next, a general linear model was applied to the data using MATLAB to calculate significance maps. A field sign map for the flattened occipital cortex was created by defining neighboring regions with reversed phases at a functional threshold of *p* < 0.05. The field sign and phase encoding data were overlaid on the flattened occipital cortex to visualize the retinotopic maps. Using the field sign changes and phase changes as guides, the borders between the visual areas were manually outlined. Figure 3 illustrates the delineation of V1, dV2, and vV2 regions.

#### ROI localizer for the crowding task

The preprocessing of the functional data for outlining tROIs was performed using the FsFast (FreeSurfer Functional Analysis Stream) tool, integrated within the FreeSurfer software package. The preprocessing steps included motion correction through rigid-body transformation onto a reference volume, brain-mask creation, and intensity normalization. The functional data for labeling tROIs were spatially smoothed with a kernel size of 5 mm. The functional images were subsequently registered to the corresponding anatomical brain images of each individual subject.

The tROI was defined as an area of the cortex that contained voxel clusters that responded to crowding stimuli during ROI localizer scan sessions. A general linear model was used to calculate the statistical parametric maps. Preprocessed data were convolved based on the assumption that the hemodynamic response function followed a cumulative gamma function. The parameters were set to δ = 2.25 seconds, τ = 1.25 seconds, and α = 2. The fixation blocks, in which only the fixation cross was presented, were then contrasted against the stimulation blocks. The resulting significance maps were overlaid on the inflated cortical surface of the left hemisphere to visualize the BOLD activity, and the ROI was defined manually. The resulting significance maps were superimposed on the inflated cortical surface of the left hemisphere to visualize the BOLD activity, and the tROI was manually defined. Significance maps were adjusted to a threshold of *p* ≤ 0.001. Figure 3A illustrates the tROI of one representative subject overlaid on the left hemisphere.

#### pRF data

Preprocessing of pRF data and model fitting were performed by using FreeSurfer 6.0 and the SamSrf 7 toolbox for pRF mapping, respectively. The processing stream followed the published protocol (Schwarzkopf, 2018). The preprocessing of pRF data was carried out similarly as for the tROI data processing. However, spatial smoothing was omitted, as the latter could potentially compromise the quality of the data (Schwarzkopf, 2018). The steps involved averaging of all runs, motion correction, brain mask creation, and applying intensity normalization. Subsequently, the pRF was modeled using a two-dimensional Gaussian. Three parameters of a symmetrical, two-dimensional Gaussian pRF model were estimated for each voxel independently: *x*0, *y*0, and σ, where the first two represent the center coordinates of the pRF in the visual field and σ is the estimate of pRF size. The model predicted the neural response at each time point of the fMRI time course from the overlap between the pRF model and a binary mask of the visual stimulus; the resulting time course was then convolved with a canonical hemodynamic response function.

The model scaling factor and the eccentricity range were, respectively, set to a radius of 9°. pRF model fitting was conducted in two stages. First, a coarse fit was conducted by using a brute force grid search on model parameters (*x*0, *y*0, and σ). Then, we identified the combination of pRF parameters that best predicted the measured time course. Various descriptions of the data were then derived from these parameters, including polar angle, eccentricity, and amount of explained variance *R*^2^. Next, we conducted a fine fit by using parameters identified by the coarse fit to seed an optimization algorithm on a vertex-by-vertex basis (Lagarias, Reeds, Wright, & Wright, 2006; Nelder & Mead, 1965) to minimize the sum of squared residuals between the predicted and observed time course. *R*^2^ for the field sign was set to 0.05; therefore, only voxels whose pRF model could explain at least 5% of the variance of the raw data were included for further analyses.

Following model fitting, the functional data for each ROI (dV1, vV1, dV2, vV2, and tROI) underwent expansion to construct a surface structure. Additionally, the anatomical surface meshes were incorporated into this structure. To estimate pRF size changes as a function of eccentricity, we grouped the voxels into 0.5° bins, starting from the parafoveal region (0.5°) up to 9° of eccentricity for ROIs dV1, vV1, dV2, and vV2.

### Statistical analysis

The statistical analyses were conducted using SPSS Statistics 28 (IBM, Chicago, IL). Due to the repeated-measures design, paired-sample *t*-tests were performed instead of independent-sample *t*-tests. In cases where the distribution assumptions for the *t*-tests were not fully met, the tests were performed using the bootstrapping method (Efron, 2007; Efron & Tibshirani, 1994). This approach involves drawing a predetermined number of random bootstrap samples with replacement from the existing sample to estimate the distribution of the data samples being compared. The sample sizes were set to 10,000 samples. The bootstrapped distributions were used to calculate bias-corrected and accelerated 95% confidence intervals (95% BCa-CIs) for the *t*-values. In situations where the assumptions for computing correlation coefficients were not met, we provide 95% BCa-CIs for the *r*-values in brackets. Effect sizes are reported based on Cohen (1988).

Power analyses were performed using the software G*Power (Erdfelder, Faul, Buchner, & Lang, 2009; Faul, Erdfelder, Lang, & Buchner, 2007) to estimate the power achieved in the performed statistical analyses. All power analyses were performed using two-tailed testing, and the criterion of 0.80 for sufficient power was adapted from Cohen (2013). Nonsphericity correction ε was calculated according to Greenhouse–Geisser for the post hoc analyses on the *F*-tests.

#### Psychophysical data

The analysis of the psychophysical data followed a process similar to that used by Malania and colleagues (2020). The “critical spacing” in the psychophysical experiments was defined as the target-to-flanker spacing corresponding to 68% correct target identification. This calculation was performed for each subject and for both flanker orientations.

To assess the training effect on the critical spacing, a crowding index was computed for each day of training for tangential and radial flankers (*C_t_* and *C_r_*, respectively). This crowding index represents the average critical spacing for each subject on the respective training day. Additionally, a crowding anisotropy index (*C_a_*) was calculated for each day of training. This index quantifies the ratio of the difference in critical spacing (in degrees of visual angle) between the radial (*C_r_*) and tangential (*C_t_*) flanker conditions to the sum of critical spacing in both conditions:

(1) $$\begin{array}{c} C_{a}=\;\frac{\left(C_{r}-C_{t}\right)}{\left(C_{r}+C_{t}\right)}\; \end{array}$$

The effect of the flanker configuration and the training days on the average critical spacing was examined. Therefore, after testing for homoscedasticity, a two-way repeated-measures analysis of variance (rmANOVA) with a 2 (flanker orientation: radial vs. tangential) × 4 (day of training: 1 vs. 2 vs. 3 vs. 4) design was performed. Additionally, two paired-sample *t-*tests were conducted to evaluate potential significant differences in critical spacing between day 1 (pre-training condition) and day 4 (post-training condition) for both radial and tangential flanker configurations. Another paired-sample *t*-test was conducted to test whether the anisotropy index (*C_a_*) significantly differed between the first and the last day of the training.

#### Analysis of the pRF parameters

rmANOVAs and paired-samples *t*-tests were applied to assess the impact of training and ROI on pRF sizes, eccentricity of the pRF, and polar angle. Prior to conducting the parametric tests, we determined whether the pre-to-post differences were normally distributed and checked for the presence of outliers. The data met both of the normality assumptions and did not contain any outliers. Consequently, we conducted paired-sample *t*-tests comparing pre- and post-training for pRF sizes in our ROIs.

## Results

### Psychophysical data

**Figure 4.**
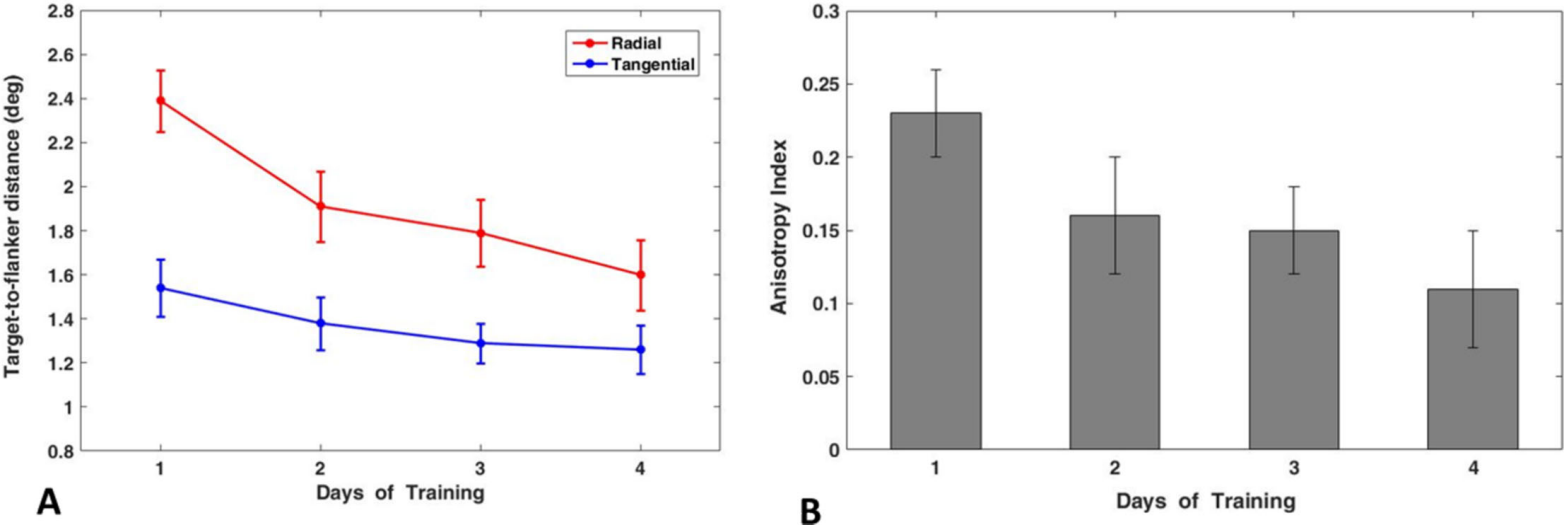
Results of behavioral training. (**A**) Changes in critical spacing measurements over the course of the 4-day training period for both radial and tangential flanker configurations. There was a significant reduction of critical spacings for both flanker configurations. (B) Mean values of the crowding anisotropy indices (*C_a_*) calculated for each of the 4 days of training. *C_a_* declined significantly after training (*p* = 0.004).

On average, the mean critical spacings were higher for the radial flanker orientation compared to the tangential flanker orientation. Furthermore, the mean critical spacings decreased with each day of the training for both flanker conditions (see Figure 4A). On day 1 (pre-training), the critical spacing was *M* = 2.39 (*SD* = 0.53) for the radial and *M* = 1.54 (*SD* = 0.49) for the tangential condition. The lowest values were observed on day 4 (post-training), with *M* = 1.60 (*SD* = 0.62) for the radial condition and *M* = 1.26 (*SD* = 0.43) for the tangential condition.

To examine the impact of training on the critical spacing (i.e., on the strength of the crowding effect), a two-way rmANOVA was performed with the factors flanker configuration (radial vs. tangential) and day of training (1 vs. 2 vs. 3 vs. 4). Normality was confirmed using the Shapiro–Wilk test, and no outliers were found. Mauchly’s test indicated violations of sphericity for the interaction between flanker configuration and days of training (each *p* = 0.044). Given the sample size of 15 subjects, which is considered a small sample size, the Greenhouse–Geisser correction was applied to correct for violations of sphericity as suggested by Girden (1992). The rmANOVA revealed a statistically significant difference between radial and tangential flanker configurations, *F*(1, 14) = 25.11, *p* < 0.001, partial η^2^ = 0.64. Furthermore, there was a significant difference in mean critical spacings across the days of training, *F*(2.05, 28.75) = 38.54, *p* < 0.001, partial η^2^ = 0.73. Additionally, the interaction between flanker configuration and days of training was significant, *F*(2.17, 30.44) = 7.79, *p* = 0.001, partial η^2^ = 0.36, indicating a significant reduction in the anisotropy of crowding.

Paired-samples *t*-tests were conducted to evaluate the differences in critical spacing between day 1 (pre-training measurements) and day 4 (post-training measurements) of training in each flanker configuration. The differences were found to be normally distributed (radial condition: *p* = 0.183; tangential condition: *p* = 0.621) according to the Shapiro–Wilk test. The *t*-tests indicated a significant reduction in critical spacing from day 1 to day 4 for both the radial condition, *t*(14) = 7.27, *p* < 0.001, Cohen's *d* = 1.88, and the tangential condition, *t*(14) = 3.69, *p* = 0.001, Cohen's *d* = 0.95.

The anisotropy index (*C_a_*) declined throughout the training. The highest mean value was observed on day one *M* = 0.23 (*SD* = 0.12). The lowest mean anisotropy index was observed on day 4 (*M* = 0.11, *SD* = 0.15). The mean *C_a_* indices for each day of training are depicted in Figure 4B. The Shapiro–Wilk test indicated no significant deviation from a normal distribution (*p* = 0.138) for anisotropy indices. A paired-sample *t*-test showed a significant reduction in anisotropy from day 1 to day 4 of training, *t*(14) = 3.05, *p* = 0.004, Cohen's *d* = 0.79.

### pRF estimates

**Figure 5.**
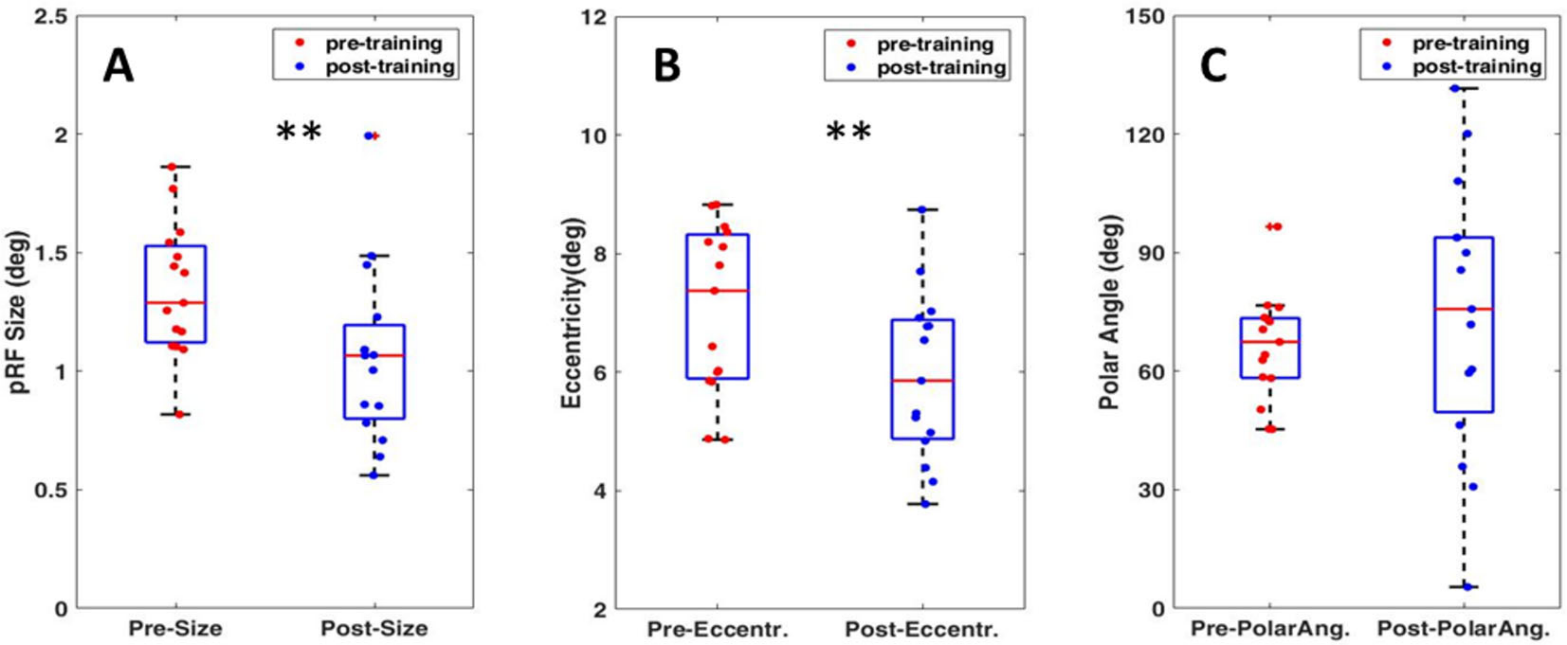
pRF parameter estimates from tROI. (**A**) Demonstration of pRF size comparisons in tROI in pre- and post-training measurements as a box-and-whisker plot. Altogether pRF size was reduced by 21% in tROI after training. (**B**, **C**) Eccentricity (**B**) and polar angle (**C**) measures in pre- and post-training conditions.

For each subject, we extracted the mean pRF size from the tROI in both pre- and post-training conditions and conducted a paired-sample *t*-test. Post-training pRF size was decreased significantly compared to pre-training pRF size, *t*(14), *p* = 0.005, Cohen's *d* = 0.87. To minimize the effect of artifacts, we filtered the data by excluding pRF sizes with negative beta values. Simultaneously, we assessed the goodness of the fit and changes in polar angle and eccentricity within the tROI. The goodness of the fit worsened slightly after training (mean pre-training *R*^2^ = 0.338, post-training *R*^2^ = 0.317). A significant shift of eccentricity toward the fovea was observed (mean pre-training eccentricity = 7.05°, post-training eccentricity = 5.9°), *t*(14), *p* = 0.006, Cohen's *d* = 0.84. Polar angle showed no significant changes after training (mean pre-training polar angle = 66.07°, post-training polar angle = 73.92°), *t*(14), *p* = 0.36, Cohen's *d* = –0.24. A comparison of pRF size measures within the tROI for pre- and post-training conditions is presented in Figure 5A, and the results indicate a significant reduction in the pRF size. At the same time, the estimated pRF centers shifted toward the fovea (Figure 5B). We did not find any significant correlation between change in the extent of critical spacing (both radial and tangential directions) and pRF sizes, *r*(15) = 0.198 and *p* = 0.480 for the tangential direction, and *r*(15) = 0.351 and *p* = 0.200 for the radial direction.

**Figure 6.**
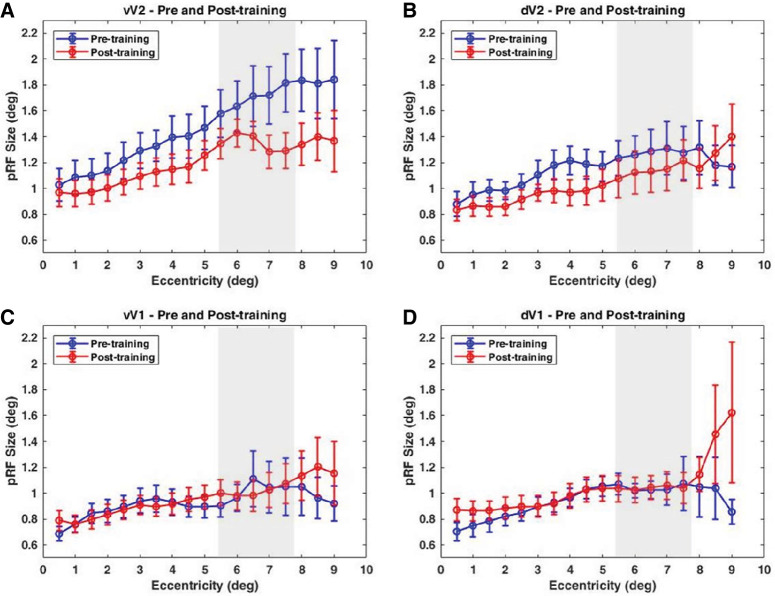
Mean pRF sizes binned into eccentricity bands of 0.5° width. (**A**, **B**) pRF sizes are plotted as a function of eccentricity in ventral and dorsal V2. (**C**, **D**) pRF sizes are plotted along eccentricities in ventral and dorsal V1. Blue curves correspond to pre-training measurements, and red curves correspond to post-training estimates of pRF sizes. The gray vertical bar indicates the visual field area where the crowding stimuli were presented during training on the crowding task. Error bars represent ±1 *SE*. Results are presented for the right hemisphere (contralateral to trained hemifield).

To explore how training on the crowding task modulates neurons at the different visual cortex levels, we compared pRF sizes in dorsal and ventral V1 and V2. As mentioned earlier, voxels were binned in 0.5° bins and mapped against eccentricity (Figure 6). rmANOVA with the factors ROI spatial location (ventral vs. dorsal), ROI (V1, V2), and training (pre-/post-) was conducted. Test results revealed a significant effect of ROI spatial location, *F*(1, 17) = 78.87, *p* < 0.001, partial η^2^ = 0.823; ROI type, *F*(1, 17) = 214.05, *p* < 0.001, partial η^2^ = 0.926; and training, *F*(1, 17) = 8.7, *p* = 0.009, partial η^2^ = 0.339. The results of the paired-sample *t*-tests demonstrated a significant change in mean pRF size for voxels belonging to vV2 (mean pre-training pRF size = 1.47°, post-training pRF size = 1.2°), *t*(17), *p* < 0.0000001, Cohen's *d* = 0.14, and dV2 (mean pre-training pRF size = 1.15°, post-training pRF size = 1.04°), *t*(17), *p* = 0.009, Cohen's *d* = 0.11). No significant change of the pRF sizes was observed in vV1 (mean pre-training pRF size = 0.93°, post-training pRFsize = 0.96°), *t*(17), *p* = 0.181, Cohen's *d* = 0.1, or dV1 (mean pre-training pRF size = 0.94°, post-training pRF size = 1.04°), *t*(17), *p* = 0.06, Cohen's *d* = 0.2. If anything, dV1 exhibited a slight increase of pRF sizes for large eccentricities, but this effect was not significant.

Although V2 exhibited a significant reduction in pRF sizes, the correlation coefficients between pRF sizes and critical spacing across subjects were not significant for both radial, *r*(15) = 0.243, *p* = 0.38 and *r*(15) = 0.118, *p* = 0.676, and tangential flanker configurations, *r*(15) = 0.268 and *p* = 0.33 for the radial direction, and *r*(15) = 0.021 and *p* = 0.94 flanker configurations. Similarly, no significant correlation was found between pRF sizes in vV1 and dV1 and critical spacing changes in both radial and tangential directions.

## Discussion

The primary objective of this study was to explore how practice on a crowding task modulates pRF properties in the early visual cortex. By examining these processes, we aimed to uncover the neural mechanisms underlying the performance improvement in a crowding task following training. The specific plastic changes occurring in neuronal networks contributing to the performance improvement in the crowding task remain not fully understood.

The initial evidence for receptive field plasticity originated from seminal studies on retinal lesions, which reported a retinotopic remapping of the visual cortex following a retinal lesion (Castaldi, Lunghi, & Morrone, 2020; Chino, Kaas, Smith, Langston, & Cheng, 1992; Dreher, Burke, & Calford, 2001; Gilbert & Wiesel, 1992; Kaas et al., 1990; Pettet & Gilbert, 1992; Wandell & Smirnakis, 2009). Recently, Silva et al. (2021), demonstrated a correlation between visual acuity and pRF sizes in an aging population.

Furthermore, the study conducted by He et al. (2019) revealed that weaker crowding effects were associated with smaller pRF sizes within the V2 region, specifically in the target voxels, which represent a cluster of voxels responsive to the stimuli. Preliminary observations were reported recently by Ozkirli, Jastrzębowska, Draganski, and Herzog (2021). Their study revealed increased pRF sizes in the crowding condition compared to the non-crowded condition across visual areas ranging from V1 to V4.

Our results replicated and further extended these findings by demonstrating that visual perceptual training can alter properties of the peripheral visual field, leading to modifications in corresponding neural representations within the visual cortex. Following training, the peripheral visual field location exhibits reduced anisotropy of the crowding zone. Specifically, we found that the critical spacing for both radial and tangential flanker configurations significantly decreased. Crowding anisotropy index (*C_a_*) calculations also revealed a substantial decline of about 53% between the initial and final training sessions. At the neural level, we observed a significant decrease, approximately 21%, in pRF sizes in the trained visual field loci (tROI). However, pRF size changes did not correlate with changes in crowding zone sizes across subjects. In our experiments, we assessed crowding zone changes in radial and tangential directions separately. Our findings demonstrate that the crowding zone undergoes significant shrinkage primarily in the radial axis direction; that is, the elliptical shape of the crowding zone is significantly reduced in the radial axis direction after training. It is important to note that the pRF fitting model used in our study cannot estimate changes in pRF shape, as it inherently treats pRFs as having a circular shape. This approach contrasts with the evidence that receptive fields may have an elongated shape (Hubel & Wiesel, 1962; Yoshor, Bosking, Ghose, & Maunsell, 2007). Considering this evidence, we can speculate that the absence of correlation may be related to undetected pRF shape changes. The recent study by Lerma-Usabiaga, Winawer, and Wandell (2021), which evaluated different pRF models, suggested that the actual aspect ratio for elliptical pRFs in early visual cortex may be less than 2, contrary to previously reported ratios. As they stated in their paper, existing software lacks the sensitivity required to differentiate ellipses with aspect ratios of 1.5 or less from circles. Furthermore, we hypothesize that the observed eccentricity shift toward the center in post-training measurements (cf. Figure 5B) may, to some extent, be associated with changes in pRF shape.

To further explore the neural mechanisms contributing to performance enhancement in the visual crowding task, we separately extracted the pRF parameters from dorsal and ventral segments of V1 and V2 and mapped them as a function of eccentricity. A systematic increase in pRF sizes was observed with increasing eccentricity, comparable to the previously reported pRF sizes in V1 and V2 (Dumoulin & Wandell, 2008, see their figure 8; Harvey & Dumoulin, 2011, see their Figure 4).

Similar to the findings from He et al. (2019), our study also revealed a significant reduction in pRF sizes in both dorsal and ventral V2 as a consequence of crowding task training, but no changes were detected in V1, neither in V3 nor in V4, in that study. Eccentricities beyond the tROI exhibited large differences between pre- and post-training curves in V2. The effect of training was more prominent for vV2, particularly in the section of visual field area where crowding stimuli were presented, as indicated by a small dip in the post-training curve. Voxels within vV1 and dV1 showed no significant reduction in pRF sizes in the post-training condition. Our results align well with a previous finding from He et al. (2019), who pointed to a key role of V2 neurons in improving performance on an orientation crowding task.

Several potential mechanisms can be proposed to explain enhanced visual discrimination in the trained visual field. One key viewpoint suggests that the training-induced improvements are linked to changes in the population receptive field properties. According to the receptive field theory, at greater eccentricities where receptive fields are larger, both target and flankers may fall within the same receptive field. This can cause the target and flanker features to become “jumbled,” resulting in misidentification of the target. However, training may induce a reduction in pRF sizes through the finetuning of neural circuits in response to the specific demands of the trained visual task. Consequently, it becomes less likely for the target and flanker features to interfere with each other, thereby weakening the crowding effect. Gilbert et al. (2001) reviewed the neural mechanisms of perceptual learning; they proposed that increased precision and discrimination result from the refinement of tuning curves and a reduction in the size of neuronal ensembles representing trained attributes. Gilbert and colleagues suggest that, with learning, neurons acquire greater selectivity and optimally distance themselves from each other, thereby enhancing their coverage of the stimulus domain. We agree with this suggestion, and we also hypothesize that the reduction in pRF sizes plays a role in enhancing spatial resolution and makes feature binding processes more accurate. The specific impact of V2 neurons in this process can be explained by the two-stage model of visual crowding (Levi, 2008; Pelli & Tillman, 2008). This model suggests that V2 plays a crucial role in the second stage by preventing inappropriate integration of features.

Furthermore, attentional modulation also contributes to training-related improvements in the crowding task. Visual training could enhance attentional modulation and improve the ability to selectively attend to relevant visual stimuli. Plastic changes in pRF size may contribute to this effect by allowing more precise spatial tuning of neurons to the attended target, effectively reducing the receptive field size and improving the discrimination of fine details (He et al., 2019; He, Cavanagh, & Intriligator, 1997; Intriligator & Cavanagh, 2001; Strasburger, 2005; Strasburger & Malania, 2013; Sundberg, Mitchell, & Reynolds, 2009). These two hypotheses are not mutually exclusive but rather complement each other to explain the training-induced pRF modulation in the crowding task.

Additional explanation could be related to GABAergic inhibition. Gamma-aminobutyric acid (GABA) is an inhibitory neurotransmitter that plays a crucial role in regulating neural activity and plasticity (Frank et al., 2022; Hoshino, Zheng, & Watanabe, 2018). Visual training may affect the balance of excitation and inhibition within the visual cortex, leading to changes in pRF properties. Modulations in GABAergic inhibition may influence the receptive field sizes and shapes of the neuron (Alitto & Dan, 2010).

### Limitations of the present study

It is important to acknowledge the limitations of our study. The relatively small sample size may limit the generalizability of the findings, and further replication in a larger sample is necessary. Another limitation of this study is related to the absence of eye movement recordings to exclude the contribution of fixation stability in the pRF size changes. We encouraged participants to maintain central fixation by providing a demanding central fixation task during fMRI recordings. We checked the performance on the central fixation task and found that the hit rate was about 95%. We cannot rule out the impact of a fixation shift, but we still can conclude that the overwhelming majority of our participants exhibited stable fixation. We also point to a recent study (Raveendran, Krishnan, & Thompson, 2020) where the authors claim that there is no association between reduced fixation stability and crowding. Nevertheless, the absence of eye movement recordings during fMRI runs poses a limitation. Although the majority of subjects demonstrated stable fixation, the impact of fixation shift cannot be completely ruled out. Last but not least, we suggest that the use of more biologically plausible models, capable of detecting pRF shape properties, would yield more complete information regarding pRF modifications following training.

## Conclusions

Prolonged training of an eccentric visual discrimination task, as implemented in the present study in the form of a gap detection task with flanked Landolt C optotypes, leads to a significant reduction in crowding and a reduction in the radial-tangential anisotropy of crowding. This reduction in the visual crowding effect, determined through psychophysical measures, coincided with a significant decrease of estimated sizes of the pRFs within the tROI and V2, particularly in the trained visual loci. These findings suggest that the early visual cortex exhibits neural plasticity in response to training in eccentric vision, supporting the idea that pRF size is a critical factor in determining the strength of crowding. Our findings indicate a potential association between changes in pRF properties and enhanced performance on visual tasks following training. They also support the two-stage model of visual crowding, emphasizing the significant role of V2 neurons in crowding-task improvement.

Our study contributes to the broader understanding of how the brain adapts and optimizes in response to training. Such knowledge can have implications for developing interventions aimed to enhance visual perception in individuals with visual impairments. Furthermore, our findings have practical implications for the improvement of targeted rehabilitation strategies. By gaining insights into the specific mechanisms underlying brain plasticity, we can tailor rehabilitation protocols to address these mechanisms and effectively improve visual perception in clinical settings. This may be particularly relevant for developing visual rehabilitation protocols that specifically aim to improve patients’ ability to use eccentric vision, which is often compromised in cases of retinal diseases such as age-related macular degeneration.

## References

[bib1] Ahmadi, K., Fracasso, A., Puzniak, R. J., Gouws, A. D., Yakupov, R., Speck, O., … Hoffmann, M. B. (2020). Triple visual hemifield maps in a case of optic chiasm hypoplasia. *NeuroImage,* 215, 116822, 10.1016/j.neuroimage.2020.116822. 32276070

[bib2] Alitto, H. J., & Dan, Y. (2010). Function of inhibition in visual cortical processing. *Current Opinion in Neurobiology,* 20(3), 340, 10.1016/j.conb.2010.02.012. 20307968 PMC3572778

[bib3] Altman, J., & Das, G. D. (1965). Autoradiographic and histological evidence of postnatal hippocampal neurogenesis in rats. *The Journal of Comparative Neurology,* 124(3), 319–335, 10.1002/cne.901240303. 5861717

[bib4] Alvarez, I., Smittenaar, R., Handley, S. E., Liasis, A., Sereno, M. I., Schwarzkopf, D. S., & Clark, C. A. (2020). Altered visual population receptive fields in human albinism. *Cortex,* 128, 107–123, 10.1016/j.cortex.2020.03.016. 32334151

[bib5] Baylor, D. A. (1987). Photoreceptor signals and vision. Proctor lecture. *Investigative Ophthalmology & Visual Science,* 28(1), 34–49. 3026986

[bib6] Berardi, N., Pizzorusso, T., & Maffei, L. (2000). Critical periods during sensory development. *Current Opinion in Neurobiology,* 10(1), 138–145, 10.1016/S0959-4388(99)00047-1. 10679428

[bib7] Beyeler, M., Rokem, A., Boynton, G. M., & Fine, I. (2017). Learning to see again: Biological constraints on cortical plasticity and the implications for sight restoration technologies. *Journal of Neural Engineering,* 14(5), 051003, 10.1088/1741-2552/AA795E. 28612755 PMC5953572

[bib8] Bouma, H. (1973). Visual interference in the parafoveal recognition of initial and final letters of words. *Vision Research,* 13(4), 767–782, 10.1016/0042-6989(73)90041-2. 4706350

[bib9] Castaldi, E., Lunghi, C., & Morrone, M. C. (2020). Neuroplasticity in adult human visual cortex. *Neuroscience & Biobehavioral Reviews,* 112, 542–552, 10.1016/j.neubiorev.2020.02.028. 32092315

[bib10] Chino, Y. M., Kaas, J. H., Smith, E. L., Langston, A. L., & Cheng, H. (1992). Rapid reorganization of cortical maps in adult cats following restricted deafferentation in retina. *Vision Research,* 32(5), 789–796, 10.1016/0042-6989(92)90021-a. 1604848

[bib11] Chung, S. T. L. (2007). Learning to identify crowded letters: Does it improve reading speed? *Vision Research,* 47(25), 3150–3159, 10.1016/j.visres.2007.08.017. 17928026 PMC2134936

[bib12] Cohen, J. (1988). Statistical power analysis for the behavioral sciences (2nd ed.). New York: Routledge.

[bib13] Cohen, J. (2013). Statistical *power analysis for the behavioral sciences* (Revised ed.). New York: Routledge.

[bib14] De Best, P. B., Raz, N., Guy, N., Ben-Hur, T., Dumoulin, S. O., Pertzov, Y., ... Levin, N. (2019). Role of population receptive field size in complex visual dysfunctions: A posterior cortical atrophy model. *JAMA Neurology,* 76(11), 1391–1396, 10.1001/jamaneurol.2019.2447. 31403655 PMC6692840

[bib15] Dekker, T. M., Schwarzkopf, D. S., de Haas, B., Nardini, M., & Sereno, M. I. (2019). Population receptive field tuning properties of visual cortex during childhood. *Developmental Cognitive Neuroscience,* 37, 100614, 10.1016/j.dcn.2019.01.001. 30777677 PMC6969313

[bib16] Desikan, R. S., Ségonne, F., Fischl, B., Quinn, B. T., Dickerson, B. C., Blacker, D., … Killiany, R. J. (2006). An automated labeling system for subdividing the human cerebral cortex on MRI scans into gyral based regions of interest. *NeuroImage,* 31(3), 968–980, 10.1016/j.neuroimage.2006.01.021. 16530430

[bib17] DeYoe, E. A., Carman, G. J., Bandettini, P., Glickman, S., Wieser, J., Cox, R., … Neitz, J. (1996). Mapping striate and extrastriate visual areas in human cerebral cortex. *Proceedings of the National Academy of Sciences, USA,* 93(6), 2382–2386, 10.1073/pnas.93.6.2382. PMC39805 8637882

[bib18] Dosher, B., & Lu, Z. L. (2017). Visual perceptual learning and models. *Annual Review of Vision Science,* 3, 343–363, 10.1146/annurev-vision-102016-061249. PMC6691499 28723311

[bib19] Dreher, B., Burke, W., & Calford, M. B. (2001). Cortical plasticity revealed by circumscribed retinal lesions or artificial scotomas. *Progress in Brain Research,* 134, 217–246, 10.1016/S0079-6123(01)34016-5. 11702546

[bib20] Dumoulin, S. O., & Knapen, T. (2018). How visual cortical organization is altered by ophthalmologic and neurologic disorders, *Annual Review of Vision Science,* 4, 357–379, 10.1146/annurev-vision-091517-033948. 29889657

[bib21] Dumoulin, S. O., & Wandell, B. A. (2008). Population receptive field estimates in human visual cortex. *NeuroImage,* 39(2), 647–660, 10.1016/j.neuroimage.2007.09.034. 17977024 PMC3073038

[bib22] Dunn, F. A., Lankheet, M. J., & Rieke, F. (2007). Light adaptation in cone vision involves switching between receptor and post-receptor sites. *Nature,* 449(7162), 603–606, 10.1038/nature06150. 17851533

[bib23] Efron, B. (2007). Bootstrap methods: Another look at the jackknife. *The Annals of Statistics,* 7(1), 1–26, 10.1214/aos/1176344552.

[bib24] Efron, B., & Tibshirani, R. J. (1994). An *introduction to the bootstrap*. London: Chapman & Hall.

[bib25] Engel, S. A., Rumelhart, D. E., Wandell, B. A., Lee, A. T., Glover, G. H., Chichilnisky, E. J., ... Shadlen, M. N. (1994). fMRI of human visual cortex. *Nature,* 369(6481), 525, 10.1038/369525A0. 8031403

[bib26] Erdfelder, E., Faul, F., Buchner, A., & Lang, A. G. (2009). Statistical power analyses using G*Power 3.1: Tests for correlation and regression analyses. *Behavior Research Methods,* 41(4), 1149–1160, 10.3758/brm.41.4.1149/metrics. 19897823

[bib27] Espinosa, J. S., & Stryker, M. P. (2012). Development and plasticity of the primary visual cortex. *Neuron,* 75(2), 230–249, 10.1016/j.neuron.2012.06.009. 22841309 PMC3612584

[bib28] Fahle, M., & Poggio, T. (2002). *Perceptual learning*. Cambridge, MA, MIT Press.

[bib29] Faul, F., Erdfelder, E., Lang, A. G., & Buchner, A. (2007). G*Power 3: A flexible statistical power analysis program for the social, behavioral, and biomedical sciences. *Behavior Research Methods,* 39(2), 175–191, 10.3758/bf03193146/metrics. 17695343

[bib30] Fiorentini, A., & Berardi, N. (1980). Perceptual learning specific for orientation and spatial frequency. *Nature,* 287(5777), 43–44, 10.1038/287043a0. 7412873

[bib31] Fischl, B. (2012). FreeSurfer. *NeuroImage,* 62(2), 774–781, 10.1016/j.neuroimage.2012.01.021. 22248573 PMC3685476

[bib32] Fischl, B., van der Kouwe, A., Destrieux, C., Halgren, E., Ségonne, F., Salat, D. H., … Dale, A. M. (2004). Automatically parcellating the human cerebral cortex. *Cerebral Cortex,* 14(1), 11–22, 10.1093/cercor/bhg087. 14654453

[bib33] Flom, M. C., Weymouth, F. W., & Kahneman, D. (1963). Visual resolution and contour interaction. *Journal of the Optical Society of America,* 53(9), 1026, 10.1364/josa.53.001026. 14065335

[bib34] Frank, S. M., Becker, M., Qi, A., Geiger, P., Frank, U. I., Rosedahl, L. A., … Watanabe, T. (2022). Efficient learning in children with rapid GABA boosting during and after training. *Current Biology,* 32(23), 5022–5030.e7, 10.1016/j.cub.2022.10.021. 36384138

[bib35] Frank, S. M., Greenlee, M. W., & Tse, P. U. (2018). Long time no see: Enduring behavioral and neuronal changes in perceptual learning of motion trajectories 3 years after training. *Cerebral Cortex,* 28(4), 1260–1271, 10.1093/cercor/bhx039. 28334110

[bib36] Gilbert, C. D., Sigman, M., & Crist, R. E. (2001). The neural basis of perceptual learning. *Neuron,* 31(5), 681–697, 10.1016/s0896-6273(01)00424-x. 11567610

[bib37] Gilbert, C. D., & Wiesel, T. N. (1992). Receptive field dynamics in adult primary visual cortex. *Nature,* 356(6365), 150–152, 10.1038/356150a0. 1545866

[bib37a] Girden, E. R. (1992). ANOVA: Repeated measures (No. 84). Sage.

[bib38] Harvey, B. M., & Dumoulin, S. O. (2011). The relationship between cortical magnification factor and population receptive field size in human visual cortex: Constancies in cortical architecture. *The Journal of Neuroscience,* 31(38), 13604–13612, 10.1523/jneurosci.2572-11.2011. 21940451 PMC6623292

[bib39] Harvey, B. M., Dumoulin, S. O., Fracasso, A., & Paul, J. M. (2020). A network of topographic maps in human association cortex hierarchically transforms visual timing-selective responses. *Current Biology,* 30(8), 1424–1434.e6, 10.1016/j.cub.2020.01.090. 32142704 PMC7181178

[bib40] He, D., Wang, Y., & Fang, F. (2019). The critical role of V2 population receptive fields in visual orientation crowding. *Current Biology,* 29(13), 2229–2236.e3, 10.1016/j.cub.2019.05.068. 31231052

[bib41] He, S., Cavanagh, P., & Intriligator, J. (1996). Attentional resolution and the locus of visual awareness. *Nature,* 383(6598), 334–337, 10.1038/383334a0. 8848045

[bib42] He, S., Cavanagh, P., & Intriligator, J. (1997). Attentional resolution. *Trends in Cognitive Sciences,* 1(3), 115–121, 10.1016/s1364-6613(97)89058-4. 21223875

[bib43] Hoshino, O., Zheng, M., & Watanabe, K. (2018). Improved perceptual learning by control of extracellular GABA concentration by astrocytic gap junctions. *Neural Computation,* 30(1), 184–215, 10.1162/neco_a_01027. 29064786

[bib44] Hubel, D. H., & Wiesel, T. N. (1962). Receptive fields, binocular interaction and functional architecture in the cat's visual cortex. *The Journal of Physiology,* 160(1), 106, 10.1113/jphysiol.1962.sp006837. 14449617 PMC1359523

[bib45] Hubel, D. H., & Wiesel, T. N. (1965). Binocular interaction in striate cortex of kittens reared with artificial squint. *Journal of Neurophysiology,* 28(6), 1041–1059, 10.1152/jn.1965.28.6.1041. 5883731

[bib46] Hubel, D. H., Wiesel, T. N., & LeVay, S. (1977). Plasticity of ocular dominance columns in monkey striate cortex. *Philosophical Transactions of the Royal Society of London. Series B, Biological Sciences,* 278(961), 377–409, 10.1098/rstb.1977.0050. 19791

[bib47] Hussain, Z., Webb, B. S., Astle, A. T., & McGraw, P. V. (2012). Perceptual learning reduces crowding in amblyopia and in the normal periphery. *The Journal of Neuroscience,* 32(2), 474–480, 10.1523/jneurosci.3845-11.2012. 22238083 PMC3428833

[bib48] Intriligator, J., & Cavanagh, P. (2001). The spatial resolution of visual attention. *Cognitive Psychology,* 43(3), 171–216, 10.1006/cogp.2001.0755. 11689021

[bib49] Kaas, J. H., Krubitzer, L. A., Chino, Y. M., Langston, A. L., Polley, E. H., & Blair, N. (1990). Reorganization of retinotopic cortical maps in adult mammals after lesions of the retina. *Science,* 248(4952), 229–231, 10.1126/science.2326637. 2326637

[bib50] Klink, P. C., Chen, X., Vanduffel, W., & Roelfsema, P. R. (2021). Population receptive fields in non-human primates from whole-brain fMRI and large-scale neurophysiology in visual cortex. *eLife,* 10, e67304, 10.7554/eLife.67304. 34730515 PMC8641953

[bib51] Kwon, M., Bao, P., Millin, R., & Tjan, B. S. (2014). Radial-tangential anisotropy of crowding in the early visual areas. *Journal of Neurophysiology,* 112(10), 2413–2422, 10.1152/jn.00476.2014. 25122703 PMC4233277

[bib52] Lagarias, J. C., Reeds, J. A., Wright, M. H., & Wright, P. E. (2006). Convergence properties of the Nelder–Mead simplex method in low dimensions. *SIAM Journal on Optimization**.,* 9(1), 112–147, 10.1137/S1052623496303470.

[bib53] Lerma-Usabiaga, G., Winawer, J., & Wandell, B. A. (2021). Population receptive field shapes in early visual cortex are nearly circular. *The Journal of Neuroscience,* 41(11), 2420–2427, 10.1523/jneurosci.3052-20.2021. 33531414 PMC7984596

[bib54] Levi, D. M. (2008). Crowding—An essential bottleneck for object recognition: A mini-review. *Vision Research,* 48(5), 635–654, 10.1016/j.visres.2007.12.009. 18226828 PMC2268888

[bib55] Malania, M., Pawellek, M., Plank, T., & Greenlee, M. W. (2020). Training-induced changes in radial-tangential anisotropy of visual crowding. *Translational Vision Science & Technology,* 9(9), 1–11, 10.1167/TVST.9.9.25. PMC7442869 32879781

[bib56] Mioche, L., & Singer, W. (1989). Chronic recordings from single sites of kitten striate cortex during experience-dependent modifications of receptive-field properties. *Journal of Neurophysiology,* 62(1), 185–197, 10.1152/jn.1989.62.1.185. 2754471

[bib57] Nelder, J. A., & Mead, R. (1965). A simplex method for function minimization. *The Computer Journal,* 7(4), 308–313, 10.1093/comjnl/7.4.308.

[bib58] Ozkirli, A., Jastrzębowska, M. A., Draganski, B., & Herzog, M. H. (2021). Isolate or combine: Population receptive field size in (un)crowding. *Journal of Vision,* 21(9), 2196, 10.1167/jov.21.9.2196.

[bib59] Parkes, L., Lund, J., Angelucci, A., Solomon, J. A., & Morgan, M. (2001). Compulsory averaging of crowded orientation signals in human vision. *Nature Neuroscience,* 4(7), 739–744, 10.1038/89532. 11426231

[bib60] Pelli, D. G., Palomares, M., & Majaj, N. J. (2004). Crowding is unlike ordinary masking: Distinguishing feature integration from detection. *Journal of Vision,* 4(12), 1136–1169, 10.1167/4.12.12. 15669917

[bib61] Pelli, D. G., & Tillman, K. A. (2008). The uncrowded window of object recognition. *Nature Neuroscience,* 11(10), 1129–1135, 10.1038/nn.2187. 18828191 PMC2772078

[bib62] Pettet, M. W., & Gilbert, C. D. (1992). Dynamic changes in receptive-field size in cat primary visual cortex. *Proceedings of the National Academy of Sciences, USA,* 89(17), 8366–8370, 10.1073/pnas.89.17.8366. PMC49919 1518870

[bib63] Plank, T., Lerner, L., Tuschewski, J., Pawellek, M., Malania, M., & Greenlee, M. W. (2021). Perceptual learning of a crowding task: Effects of anisotropy and optotype. *Journal of Vision,* 21(11):13, 1–11, 10.1167/jov.21.11.13. PMC8543403 34673900

[bib64] Polat, U., & Sagi, D. (1993). Lateral interactions between spatial channels: Suppression and facilitation revealed by lateral masking experiments. *Vision Research,* 33(7), 993–999, 10.1016/0042-6989(93)90081-7. 8506641

[bib65] Poltoratski, S., Maier, A., Newton, A. T., & Tong, F. (2019). Figure-ground modulation in the human lateral geniculate nucleus is distinguishable from top-down attention. *Current Biology,* 29(12), 2051–2057.e3, 10.1016/j.cub.2019.04.068. 31178323 PMC6625759

[bib66] Raveendran, R. N., Krishnan, A. K., & Thompson, B. (2020). Reduced fixation stability induced by peripheral viewing does not contribute to crowding. *Journal of Vision,* 20(10):3, 1–13, 10.1167/jov.20.10.3. PMC7545060 33007078

[bib67] Sasaki, Y., Nanez, J. E., & Watanabe, T. (2010). Advances in visual perceptual learning and plasticity. *Nature Reviews Neuroscience,* 11(1), 53–60, 10.1038/nrn2737. 19953104 PMC2864603

[bib68] Schwarzkopf, D. S., Anderson, E. J., de Haas, B., White, S. J., & Rees, G. (2014). Larger extrastriate population receptive fields in autism spectrum disorders. *The Journal of Neuroscience,* 34(7), 2713–2724, 10.1523/jneurosci.4416-13.2014. 24523560 PMC3921434

[bib69] Schwarzkopf, D. S. (2018). SamSrf 9.5 - Matlab toolbox for pRF analysis. Charlottesville, VA: Open Science Framework, 10.17605/osf.io/2rgsm.

[bib70a] Ségonne, F., Dale, A. M., Busa, E., Glessner, M., Salat, D., Hahn, H. K., … Fischl, B. (2004). A hybrid approach to the skull stripping problem in MRI. *NeuroImage,* 22(3), 1060–1075, 10.1016/j.neuroimage.2004.03.032. 15219578

[bib70] Sereno, M. I., Dale, A. M., Reppas, J. B., Kwong, K. K., Belliveau, J. W., Brady, T. J., … Tootell, R. B. H. (1995). Borders of multiple visual areas in humans revealed by functional magnetic resonance imaging. *Science,* 268(5212), 889–893, 10.1126/science.7754376. 7754376

[bib71] Shen, L., Han, B., & de Lange, F. P. (2020). Apparent motion induces activity suppression in early visual cortex and impairs visual detection. *The Journal of Neuroscience,* 40(28), 5471–5479, 10.1523/jneurosci.0563-20.2020. 32513825 PMC7343332

[bib72] Silson, E. H., Reynolds, R. C., Kravitz, D. J., & Baker, C. I. (2018). Differential sampling of visual space in ventral and dorsal early visual cortex. *The Journal of Neuroscience,* 38(9), 2294–2303, 10.1523/JNEUROSCI.2717-17.2018. 29382711 PMC5830517

[bib73] Silva, M. F., Harvey, B. M., Jorge, L., Canário, N., Machado, F., Soares, M., … Castelo-Branco, M. (2021). Simultaneous changes in visual acuity, cortical population receptive field size, visual field map size, and retinal thickness in healthy human aging. *Brain Structure and Function,* 226(9), 2839–2853, 10.1007/S00429-021-02338-0. 34245381 PMC8541970

[bib74] Smith, A. T., Singh, K. D., Williams, A. L., & Greenlee, M. W. (2001). Estimating receptive field size from fMRI data in human striate and extrastriate visual cortex. *Cerebral Cortex,* 11(12), 1182–1190, 10.1093/cercor/11.12.1182. 11709489

[bib75] Stiles, J. (2000). Neural plasticity and cognitive development. *Developmental Neuropsychology,* 18(2), 237–272, 10.1207/S15326942DN1802_5. 11280966

[bib76] Stoll, S., Finlayson, N. J., & Schwarzkopf, D. S. (2020). Topographic signatures of global object perception in human visual cortex. *NeuroImage,* 220, 116926, 10.1016/j.neuroimage.2020.116926. 32442640 PMC7573540

[bib77] Strasburger, H. (2005). Unfocussed spatial attention underlies the crowding effect in indirect form vision. *Journal of Vision,* 5(11):8, 1024–1037, 10.1167/5.11.8. 16441200

[bib78] Strasburger, H., & Malania, M. (2013). Source confusion is a major cause of crowding. *Journal of Vision,* 13(1):24, 1–20, 10.1167/13.1.24. 23335321

[bib79] Sun, G. J., Chung, S. T. L., & Tjan, B. S. (2010). Ideal observer analysis of crowding and the reduction of crowding through learning. *Journal of Vision,* 10(5):16, 1–14, 10.1167/10.5.16. PMC3096759 20616136

[bib80] Sundberg, K. A., Mitchell, J. F., & Reynolds, J. H. (2009). Spatial attention modulates center-surround interactions in macaque visual area V4. *Neuron,* 61(6), 952–963, 10.1016/J.NEURON.2009.02.023. 19324003 PMC3117898

[bib81] Talairach, J., & Tournoux, P. (1988). *Co-planar stereotaxic atlas of the human brain: 3-dimensional proportional system: An approach to cerebral imaging*. Stuttgart: George Thieme Verlag.

[bib82] Toet, A., & Levi, D. M. (1992). The two-dimensional shape of spatial interaction zones in the parafovea. *Vision Research,* 32(7), 1349–1357, 10.1016/0042-6989(92)90227-a. 1455707

[bib83] Wade, A. R., & Wandell, B. A. (2002). Chromatic light adaptation measured using functional magnetic resonance imaging. *The Journal of Neuroscience,* 22(18), 8148–8157, 10.1523/jneurosci.22-18-08148.2002. 12223569 PMC6758099

[bib84] Wandell, B. A., & Smirnakis, S. M. (2009). Plasticity and stability of visual field maps in adult primary visual cortex. *Nature Reviews Neuroscience,* 10(12), 873–884, 10.1038/nrn2741. 19904279 PMC2895763

[bib85] Watanabe, T., & Sasaki, Y. (2015). Perceptual learning: Toward a comprehensive theory. *Annual Review of Psychology,* 66, 197–221, 10.1146/annurev-psych-010814-015214. PMC4286445 25251494

[bib86] Welbourne, L. E., Morland, A. B., & Wade, A. R. (2018). Population receptive field (pRF) measurements of chromatic responses in human visual cortex using fMRI. *NeuroImage,* 167, 84–94, 10.1016/j.neuroimage.2017.11.022. 29155081 PMC5854267

[bib87] Whitney, D., & Levi, D. M. (2011). Visual crowding: A fundamental limit on conscious perception and object recognition. *Trends in Cognitive Sciences,* 15(4), 160–168, 10.1016/j.tics.2011.02.005. 21420894 PMC3070834

[bib88] Wolford, G., Marchak, F., & Hughes, H. (1988). Practice effects in backward masking. *Journal of Experimental Psychology. Human Perception and Performance,* 14(1), 101–112, 10.1037//0096-1523.14.1.101. 2964500

[bib89] Yoshor, D., Bosking, W. H., Ghose, G. M., & Maunsell, J. H. R. (2007). Receptive fields in human visual cortex mapped with surface electrodes. *Cerebral Cortex,* 17(10), 2293–2302, 10.1093/cercor/bhl138. 17172632

